# The Difference in Performance and Compatibility between Crystalline and Amorphous Fillers in Mixed Matrix Membranes for Gas Separation (MMMs)

**DOI:** 10.3390/polym15132951

**Published:** 2023-07-05

**Authors:** Mariolino Carta, Ariana R. Antonangelo, Johannes Carolus Jansen, Mariagiulia Longo

**Affiliations:** 1 Department of Chemistry, Faculty of Science and Engineering, Swansea University, Grove Building, Singleton Park, Swansea SA2 8PP, UK; a.antonangelo@gmail.com; 2 Institute on Membrane Technology, National Research Council of Italy (CNR-ITM), Via P. Bucci 17/C, 87036 Rende, Italy; m.longo@itm.cnr.it

**Keywords:** MOFs, PIMs, porous polymers, amorphous polymers, mixed matrix membranes, gas separation, gas transport properties

## Abstract

An increasing number of high-performing gas separation membranes is reported almost on a daily basis, yet only a few of them have reached commercialisation while the rest are still considered pure research outcomes. This is often attributable to a rapid change in the performance of these separation systems over a relatively short time. A common approach to address this issue is the development of mixed matrix membranes (MMMs). These hybrid systems typically utilise either crystalline or amorphous additives, so-called fillers, which are incorporated into polymeric membranes at different loadings, with the aim to improve and stabilise the final gas separation performance. After a general introduction to the most relevant models to describe the transport properties in MMMs, this review intends to investigate and discuss the main advantages and disadvantages derived from the inclusion of fillers of different morphologies. Particular emphasis will be given to the study of the compatibility at the interface between the filler and the matrix created by the two different classes of additives, the inorganic and crystalline fillers vs. their organic and amorphous counterparts. It will conclude with a brief summary of the main findings.

## 1. Introduction

The improvement of separation processes is crucial for the production and purification of commercially important gases, which may come either from natural sources or chemical industries [1,2,3,4]. In this context, the utilisation of membranes is acknowledged as one of the fastest-growing techniques that help to bring down the costs without losing separation performance and final purity [5,6,7,8,9]. This is especially true if we compare the costs of membranes with more traditional techniques, such as liquefaction, cryogenic distillation and various adsorption processes (i.e., Pressure swing adsorption (PSA) and Transient Absorption Spectroscopy (TAS)) [10,11,12,13]. The performance of gas separation is measured by evaluating the permeability (flux rate) of the fastest gas X in a mixture (P_x_, typically reported in Barrer or gas permeation unit (GPU), where 1 Barrer = 1 cm^3^ _STP_ cm cm^−2^ s^−1^ cmHg^−1^ and 1 GPU = 1 Barrer µm^−1^), and its purity, the selectivity (α = P_x_/P_y_) over a slower gas Y. These parameters are strongly correlated with one another, and a well-established trade-off exists between the two, meaning that if the permeability increases the selectivity decreases, and vice versa. In energy terms, this means that good membranes for gas separation should possess either a high permeability so that the separation will be rapid and a large amount of gas can be treated at once, or good selectivity so that the separation will need only one or few stages to achieve the target purity. Despite the fact that the latter may confer a more efficient separation, the gas fluxes are much lower and so a higher membrane area is needed for the same amount of gas to be treated, which increases the overall costs of the process. An empirical evaluation of the permeability vs. selectivity trade-off was first proposed by Robeson who, for the first time in 1991, gathered the best-performing materials for gas separation and plotted their permselectivity data in double logarithmic plots for selected and commercially important gas pairs [14]. He then drew a series of so-called upper bounds, stating that materials that surpassed these empirical lines possessed exceptional gas separation properties. His work was updated in 2008 [15] when he decided to introduce more polymers that systematically exceeded his previous limits. Since then, more researchers have conducted studies and gathered more data to set the separation bars always higher, suggesting newer potential upper bounds. For instance, Pinnau et al., who proposed new limits for O_2_/N_2_, H_2_/N_2_ and H_2_/CH_4_ in 2015 [16], or McKeown and co-workers who designed new upper bounds for CO_2_-based gas pairs (CO_2_/CH_4_ and CO_2_/N_2_) [17]. The best-performing polymers, plotted in what is nowadays universally recognised as “Robeson plots”, come from new research-based materials that possess very high permeability and moderate to good selectivity. Despite great improvements in state-of-the-art separations, at the moment, the industry still prefers employing low-permeability and highly selective polymers, sacrificing a high flux for better separation. This is primarily due to their more stable performance, easy processability, good mechanical strength and, of course, lower costs compared to newer materials. In fact, commercial membranes currently used in the industry, such as Matrimid^®^, Pebax^®^ and polysulfones [18,19,20,21], display a moderate combination of permeability and selectivity, and researchers around the world are constantly proposing and developing new and more efficient membrane systems that allow them to substitute these with their advanced materials. Depending on whether high gas volumes need to be processed or whether a high gas purity is needed, research on new materials may be focused either on the gas permeability or on the permselectivity of membranes.

Nevertheless, despite the exceptional initial performance and the great potential of faster and more efficient separation promised by these new systems, most of these novel membranes have not yet been commercialised, and the lower-performing ones are still dominating the market. One of the problems that hamper the exploitation of new polymeric materials for gas separation is connected to their rapid change in performance with time, a phenomenon that is known as physical ageing [22,23]. This occurs when a polymer with an initially high fractional free volume (FFV) relaxes to a denser state (within weeks, and in some cases within days), which translates into a decrease in permeability so the pressure or the number of membrane modules needs to be constantly adjusted to maintain the same flux and to guarantee the same productivity of the industrial plant. This drop in permeability is often compensated by a commensurate increase in selectivity for several gas pairs, which still places them in favourable regions of the Robeson plots. However, the change in performance represents a significant engineering concern that, so far, has convinced the industry to keep using lower-performing but stable commercial membrane materials [24].

## 2. Mixed Matrix Membranes

An increasing number of research groups is engaged in the attempt to address both the lack of performance of commercial gas separation membranes and the ageing problem of novel materials, all with moderate success. The ideal membrane should, in fact, possess, at the same time, permeability high enough to grant a fast separation (high flux) and selectivity that allows reaching the required gas purities in a single process step along with stable performance over time. A potential solution to the ageing problem is represented by the preparation of mixed matrix membranes (MMMs) [25,26,27], which are hybrid materials produced by mixing a soluble polymer (the *matrix*, usually the component that suffers from ageing) and an insoluble compound, known as the *filler* [28,29]. The combination of the two is supposed to achieve the best of both worlds, improving the performance and durability of the soluble polymeric matrices and, at the same time, employing insoluble materials that are notoriously difficult to process for gas separation [30]. An appropriate choice of filler is very important, as it can potentially achieve better molecular sieving properties of non-porous membranes. In addition, organic fillers can be further functionalised to adjust the selectivity for several gases, always with the aim to improve the overall performance and stability [31,32,33]. With this approach in mind, a number of new MMMs with an increasing variety of different and exotic fillers have been prepared by well-known research groups [26,34,35].

Initially, the proposed fillers were primarily zeolites, porous silica and metal organic frameworks (MOFs) [27,36,37,38]. The latter seemed the most strategic choice as, compared to zeolites and silicas, their synthesis can be easily tuned to accommodate different functional groups in their frameworks. It was anticipated that the functionalisation of MOFs would also improve the compatibility between the matrix and the filler so they adhere more tightly to the soluble material [39,40,41]. However, MOFs still present defects in their structures that lead to a limited chemical affinity with the polymer matrix [42,43,44,45], therefore the compatibility between these crystalline fillers and the amorphous structure of the soluble matrices remains a serious issue. This point may prove to be especially important when assessing the robustness and the general mechanical properties of the MMMs, particularly when used in typically harsh industrial conditions [31]. A last point worth consideration comes from the help of solid-state NMR studies by Schmidt-Rohr et al. [46], who proved that polymer chains could fill the pores of some large MOFs. Although this can be seen as a sign of enhanced compatibility, it can also translate into the loss of some of their porosity, which would limit the gas transport and lead to a slower flux.

**Figure 1:**
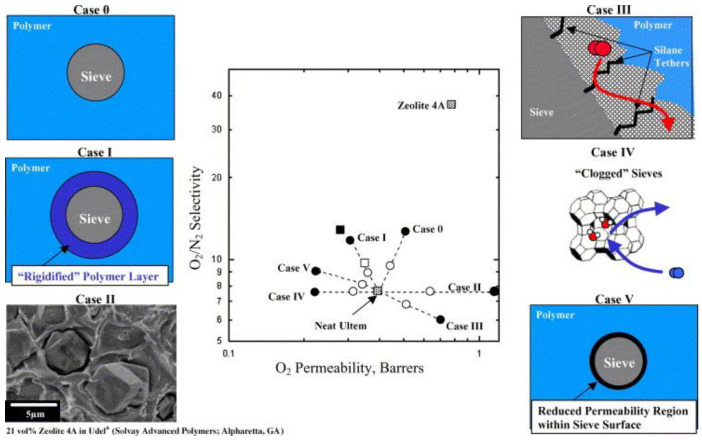
Summary of the relationship between mixed matrix membrane morphologies and transport properties. Circles represent calculated values; squares represent experimental data in Ultem^®^. Solid markers are 35 vol% zeolite 4A; open markers are 15 vol% 4A [47]. Reprinted with permission from Elsevier.

Moore and Koros [47] discussed six different cases of how the filler and polymer/filler interface affects the transport properties of the MMMs compared to the neat polymer (Figure 1). Although their studies referred to Zeolite 4A, similar behaviour must be expected for other fillers. Their studies highlight the enormous importance of the polymer–filler interface, as anticipated in the previous sections. The presence of a dispersed phase can affect the permeability of the matrix polymer in various ways, with a variation (increase or decrease) of either the permeability or the selectivity.

The precise effect depends on the properties of the matrix polymer and the filler, but also on the phenomena at the polymer–filler interface, which is why, for instance, ionic liquids were discussed above as possible compatibilizers (e.g., [48] Ref., Section 2.2.2). 

### 2.1. Description of the Transport in MMMs

The successful development of MMMs requires a full understanding of their transport properties, and for a comparison of different systems, it is helpful to find mathematical models that are able to quantify how the filler particle may affect the transport properties of the polymer and vice versa, or how the transport properties are affected by the operational conditions. 

The gas transport in polymeric membranes is universally described by the solution diffusion model [49], where the permeability (*P*) is the product of the solubility (*S*) and the diffusion coefficient (*D)*:

*P* = *D* × *S* (1)

#### 2.1.1. Computational Approaches

The most elementary approach to studying the transport in MMMs is by computational methods, using molecular or atomistic models. Fully atomistic models [50] are likely the most demanding after quantum-chemical models from the computational point of view, but they provide extremely useful molecular-level information, not only at the bulk level of the individual materials [51,52] but especially at the level of the matrix–filler interface. The latter is quite rare, and few computational studies have been reported on the understanding of the interfacial phenomena, for instance, between PIM-1 or PIM-EA-TB and ZIF-8 [44] and between PIM-1 and NUS-8 [53]. Such studies provide deep insight into the transport properties of MMMs and help to understand the experimental results.

#### 2.1.2. Permeation Models

The effective medium approach describes transport in a heterogeneous system as if it were homogeneous, so the solution-diffusion model (Equation (1)) can be used for its quantitative description. In this case, the various constituents of the mixture have different properties, with a single ‘effective’ parameter, such as an effective permeability or an effective diffusion coefficient. A good example is the Maxwell model [54] for transport in MMMs:

(2) $$P_{MMM}=P_{c}\left|\frac{P_{d}+2P_{c}-2\Phi_{d}\left(P_{c}-P_{d}\right)}{P_{d}+2P_{c}+\Phi_{d}\left(P_{c}-P_{d}\right)}\right|$$

where *P_MMM_* is the effective permeability coefficient. Despite its limitations (e.g., valid only for <30 vol% of spherical filler particles with good dispersion), the Maxwell model is by far the most-used model to describe the correlation between the permeability of the MMM and the permeabilities of the continuous phase (*P_c_*) and the dispersed phase (*P_d_*) and their respective volume fractions (*Φ_c_*, *Φ_d_*). For extreme cases with a relatively low-permeable filler, where *P_c_* ≫ *P_d_*, the Maxwell model reduces to:

(3) $$P_{MMM}=P_{c}\left|\frac{P_{c}-\Phi_{d}P_{c}}{P_{c}+0.5\Phi_{d}P_{c}}\right|$$

and for an extremely high-permeable filler, where *P_c_* ≪ *P_d_*, the model reduces to:

(4) $$P_{MMM}=P_{c}\left|\frac{1+2\Phi_{d}}{1-\Phi_{d}}\right|$$

**Figure 2:**
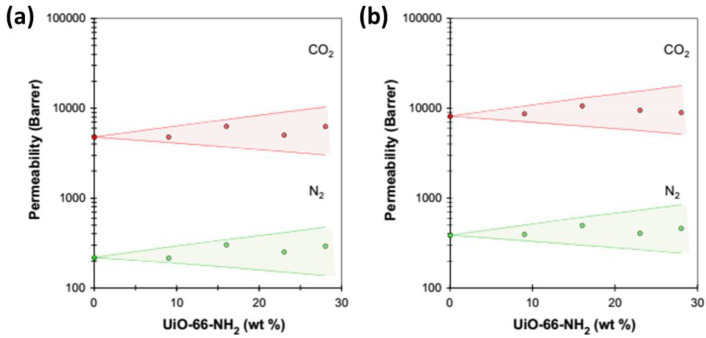
Example of the CO_2_ and N_2_ permeability of PIM-1/UiO-66-NH_2_ mixed matrix membranes as a function of the filler content, with indication of the minimum and maximum permeability limits defined by the Maxwell model [56]. (**a**) As-cast membrane and (**b**) after methanol treatment. Reprinted with permission from Elsevier.

These two cases define the maximum possible decrease in permeability (impermeable fillers) and the maximum possible increase in permeability (infinitely permeable fillers) if the matrix is not affected and there are no interface effects. Obviously, the selectivity of a system increases if the filler is more permeable than the matrix polymer for the fast-permeating species, less permeable for the slow-permeating species or a combination of both. This could be realized with crystalline fillers with a precisely designed pore structure. Alternatively, the benzimidazole-linked polymers (BILPs), seen in work by Gascon et al. [55], show an example of fillers that enhance the permeability of Matrimid^®^-based MMMs without affecting the CO_2_/N_2_ gas mixture selectivity. This indicates that only large pores were added, where both gases permeate easily. An example in which the Maxwell model describes the transport well was reported for UiO-66-(COOH)_2_ and UiO-66-NH_2_ (Figure 2) [56]. This is not the case for the 36% higher CO_2_ permeability of PIM-1 that is achieved upon the addition of only 2–5% wt of MUF-15 by Telfer et al. [57], which falls well outside the window defined by the upper and lower limits of the Maxwell model, indicating that other factors must play a role in this system and that other equations are needed.

**Figure 3:**
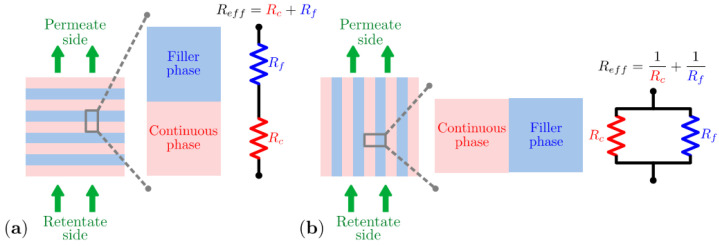
Resistance model approach for a multilayer composite in (**a**) series and (**b**) parallel [59]. Reprinted with permission from MDPI.

Much more complex equations are needed for the various situations depicted in Figure 1, where for instance interface effects between the filler and matrix play a role in the transport [47]. Furthermore, for more complex systems with non-spherical particles [58], higher filler loading, non-homogeneous dispersion, etc., various alternative models have been proposed to describe quantitatively the permeability of the MMM in terms of the individual permeability coefficients of the polymer and the filler material, their volume fractions in the mixture, the shape of the filler particles, etc. Eight different models based on Maxwell’s theory are presented in a review by Monsalve-Bravo and Bhatia [59] in which they also present an alternative to the effective medium approach, based on the resistance model approach (Figure 3). Keskin and Altinkaya provide even more detail and distinguish between six resistance-based models and twelve effective medium-based models [60].

#### 2.1.3. Sorption Models

The most commonly used model to describe gas sorption in pure polymers is the so-called dual mode sorption (DMS) model that correlates the equilibrium concentration of a gas in the polymer, *c*, with the gas pressure, *p*:

(5) $$c=c_{D}+c_{H}=k_{D}\cdot p+\frac{C_{H}^{\prime }\cdot b\cdot p}{1+b\cdot p}$$

where *c_D_* is the concentration of a dissolved gas (Henry mode) and *c_H_* is the concentration of the gas adsorbed in the “holes”. The model has three adjustable parameters, *k_D_*, the Henry’s law constant, *C^’^_H_*, the Langmuir monolayer sorption capacity and *b*, the Langmuir affinity constant. This usually very accurately describes the sorption of gases in all kinds of polymers, including microporous PIMs [61], although other models such as the Guggenheim, Anderson and de Boer (GAB) [62] may be needed to fit the adsorption of vapours or highly condensable gases, such as CO_2_ in PIMs and other high free-volume polymers [63]. The DMS model has also been successfully applied to describe the sorption in MMMs [64] and can thus be of great help to quantify the effect of the fillers not only on the permeability but also on the solubility.

This review aims to provide a critical review of the performance of mixed matrix membranes when either crystalline or amorphous materials are utilised as fillers, especially focusing on the differences in permeability, selectivity, stability at the interface, and effectiveness to reduce physical ageing. For simplicity, despite the many more crystalline fillers reported in the literature, this review will primarily discuss MOFs as crystalline fillers, especially because of their versatility from a synthesis point of view and their widespread use in the field. In addition, apart from a few interesting exceptions, all gas pairs discussed will contain CO_2_ to better compare the different MMMs from the gas separation point of view.

### 2.2. Advances in the Field of MMMs with Crystalline Fillers

Through the years, a number of different crystalline fillers has been investigated for the preparation of mixed matrix membranes, with the aim to improve the performance of the neat polymers in terms of permeability, selectivity or both. The fillers vary from fully organic cages [65,66,67,68] and covalent organic frameworks (COFs) [69,70,71,72], to COF-metal organic framework ‘alloys’ [73], pure metal organic frameworks [58], and zeolites [74]. MOFs are the largest groups and will be the focus of this work, with occasional examples of other (inorganic and crystalline) fillers.

#### 2.2.1. Improvement in Selectivity with Crystalline Fillers

**Figure 4:**
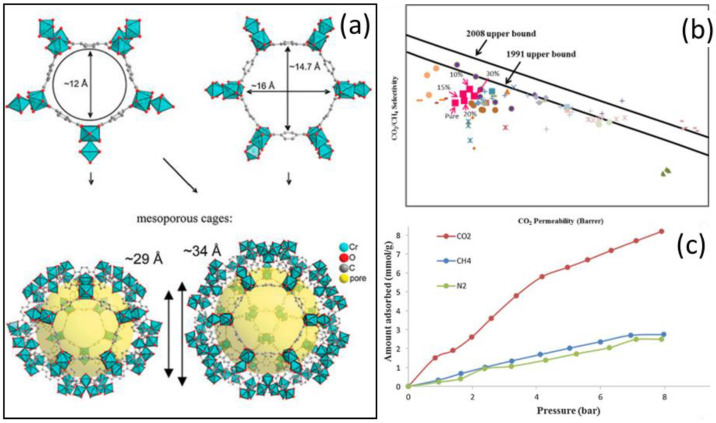
(**a**) Chemical structure of MIL-101. (**b**) Adsorption isotherms of CO_2_, CH_4_ and N_2_ for MIL-101 crystals at 25 °C. (**c**) Robeson diagrams for separation of CO_2_/CH_4_. Adapted from [75] Copyright © 2015 The Korean Society of Industrial and Engineering Chemistry. Published by Elsevier B.V. All rights reserved.

The nature of the fillers plays a crucial role when tailoring the performance of MMMs for gas separation, either by increasing the permeability for certain gases or by enhancing the selectivity to achieve a higher gas purity. For a given soluble polymer, the possibility of fine-tuning the parameters that should guarantee good separation strongly depends on the features of the insoluble additives. They are, predominantly, the porosity of the filler, the number and polarity of functional groups attached to it, and its affinity towards the soluble matrix polymer. One of the most common outcomes, derived from the preparation of new MMMs, is represented by the improvement of the selectivity for different gases for commercially available membranes. For instance, Naseri and Co-workers [75] were able to improve the selectivity of Matrimid^®^ 5218 based MMMs with the addition of up to 30% of the very porous MIL-101 (Cr) as a filler [76] (Figure 4). The neat Matrimid^®^ is a commercial low-permeability/highly selective polyimide (PI), and it shows P_CO2_ ~4.44 Barrer and P_CH4_ ~0.126 Barrer with CO_2_/CH_4_ and CO_2_/N_2_ selectivities of 35 and 34, respectively. 

Because of the much lower porosity of the matrix, compared to the filler, it would be expected that the addition of the very permeable MOF would have improved the initial low permeability, perhaps at the expense of the selectivity. Conversely, the addition of 10% wt loading of the filler produced a modest increase in P_CO2_ from ~4.44 Barrer to only ~6.95 Barrer, which was surprisingly complemented with an improvement in the selectivity of 56 for CO_2_/CH_4_ and 52 for CO_2_/N_2_ (from the starting 35 and 34 of the neat membrane) that allowed the MMM to come much closer to the 1991 upper bound for CO_2_/CH_4_ than neat Matrimid (Figure 4). A further increase in the loading produced the desired improvement of the permeability but with a reduction of the selectivity that deteriorated the overall performance. It is worth noting that the group reported that higher loadings also started producing significant defects at the polymer–filler interface.

The group of Janiak in Germany also studied selectivity improvements of Matrimid^®^ based MMMs [77], dedicating particular attention to the compatibility of their MOF filler in the membrane. They employed loadings between 4 and 24% wt of Al-fum, which was chosen for its previously reported good compatibility with Matrimid^®^ [78]. This crystalline additive was used in two different forms, a porous version with an apparent BET surface area of 1100 m^2^ g^−1^ and another one where they filled its pores with DMSO, which led to a severe reduction of its porosity (only 40 m^2^ g^−1^). The study of utilisation of the two different forms of the same MOF was specially designed to test its compatibility with Matrimid^®^, giving important insights for this review. The Scanning Electron Microscopy (SEM) images showed that, in both cases, the compatibility between the matrix and the filler was good, with microdefects appearing only after the inclusion of ~24% wt of the filler. The highest loading, surprisingly, resulted in a strong decrease in the permeability but it kept the same selectivity shown by the neat polymer. In the case of the highly porous Al-fum MMM, the authors found the best permselectivity performance using 20% wt, with P_CO2_ of ~11 Barrer and a selectivity of 60 over CH_4_, which recorded an overall 63% improvement. With the non-porous Al-fum(DMSO), they reported better adhesion of the filler, but the permeability and selectivity proved similar or even lower than the neat Matrimid^®^, with the best results showing P_CO2_ ~6 Barrer with a selectivity of ~50 over CH_4_. They concluded that, for their system, the high porosity and high fractional free volume (FFV) of the porous MOF were crucial to improving the properties of their MMMs, although the non-porous MOF produced a better adhesion.

**Figure 5:**
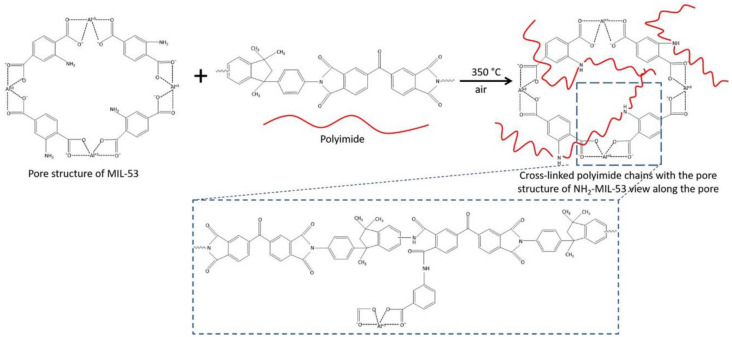
Mechanism of chemical cross-linking of Matrimid^®^ chains with the amino functions of NH_2_-MIL-53(Al) with thermal treatment at 350 °C in air. Adapted from [80] Copyright © 2020, American Chemical Society.

Vankelecom and co-workers also pushed the loading of NH_2_-MIL-53(Al) [79] up to 40% wt, in this case in an attempt to improve the performance of Matrimid^®^ [80]. The addition of the amino-containing MOF was meant to increase the known limited compatibility of the original MIL-53 with soluble polymers. To further improve it, the MMM was heated up to 350 °C to induce oxidative cross-linking and producing, what they called a PI-MOF. This experiment showed that the polyimide chains could penetrate the large pores of the MOF, which led to an outstanding improvement in the CO_2_/CH_4_ selectivity (Figure 5). They also observed that the MMMs prepared via cross-linking had a considerably improved interface compatibility between the two components, which is a crucial feature for this review. In addition to enhancing the performance for gas separation, the excellent dispersion of the filler into the matrix also improved the mechanical properties and robustness of the membrane. Conversely, morphology studies conducted on the MMMs before cross-linking did not show the same stability, somewhat confirming the limited compatibility between crystalline MOFs and amorphous polymers. The best permselectivity data of this work were achieved at 40% wt of filler loading with a 50–50% mixture of CO_2_/CH_4_, resulting in a P_CO2_ of ~5.3 Barrer (in the same range as the neat Matrimid^®^), and amazing selectivity of 153 over CH_4_ (from the starting ~76, measured in their conditions) that allowed them to approach the 2008 upper bound.

Although the improvement of the initial selectivity of low-permeability systems is crucially important to obtain high purity of the separated gases, as anticipated, low permeability translates into a low flux and, therefore, in long separation times or the impossibility to treat large gas volumes. Alternatively, large membrane areas can be used, but this is economically prohibitive. For this reason, improving the selectivity of already highly permeable polymeric membranes is of great interest. With the help of appropriate fillers, this issue can be tackled with MMMs. For example, in a recent paper, Sivaniah et al. [81] used the very permeable PIM-1 [82,83] as the matrix component for MMMs and enhanced its performances by adding 5% wt of amino-modified UiO-66 as a filler. With this amount, they reported an increase in CO_2_/N_2_ selectivity from 9.2 of the pure polymer to 24.1 of the MMM. They modulated the size of the MOF’s pores with the addition of water during its synthesis, obtaining UiO-66-NH_2_ nanocrystals of 20–30 nm in size. The dispersion of this filler into a PIM-1 solution of 5–40% wt produced mechanically robust and highly transparent MMMs. The latter is a very unusual feature as, often, the addition of insoluble additives leads to a loss in transparency instead. This confirms that the dispersion of the filler was highly homogeneous. The amino-functionalised MOF proved less porous than the original UiO-66 [84], but the increased compatibility with the organic and amorphous PIM-1 led to an enhancement of the CO_2_/N_2_ selectivity. The best results were achieved at 5–10% wt loading (P_CO2_ ~2900 Barrer α_CO2/N2_ ~27). The addition of the non-functionalised UiO-66 to PIM-1, instead, led to a higher permeability but a lower selectivity compared to the pristine PIM-1 (P_CO2_ ~3600 Barrer α_CO2/N2_ ~20). Similar results were obtained by Khdhayyer et al., who found that MMMs based on PIM-1 with neat UiO-66 or NH_2_- and COOH-functionalized UiO-66, showed CO_2_, CH_4_ and N_2_ permeabilities within the upper and lower limit of the Maxell model using up to 28% wt of filler [56], and that the increase in permeability is primarily due to an increase in the diffusion coefficient. Wang et al. also found a higher selectivity for UiO-based MMMs in 6FDA-Durene and ascribed this to an increased solubility selectivity of CO_2_/CH_4_ and the absence of interfacial defects [85]. The solubility could be well described by the dual-mode sorption model (5).

In an attempt to keep the high permeability of PIM-1 but, again, increase its selectivity for CO_2_, Coronas et al. [86] prepared MMMs by blending two soluble polymers, PIM-1 and 6FDA-DAM, and adding ZIF-8 as the inorganic filler. They started their work by seeking the best compromise between the amounts of the two soluble polymers, observing that their different solubility in chloroform (used as the casting solvent) prevented the formation of a completely homogeneous composite membrane. The best match was found at 90% of 6FDA-DAM and 10% of PIM-1. 

**Figure 6:**
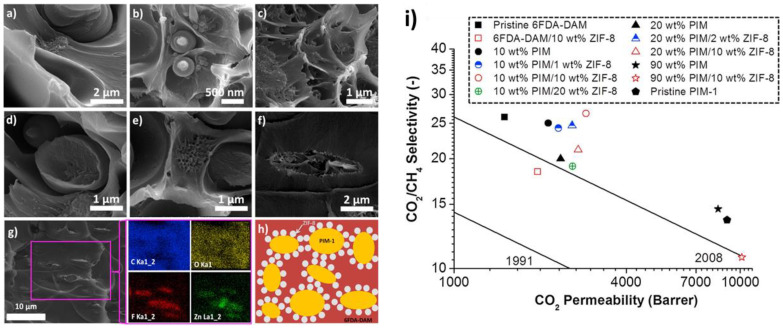
SEM images of MMMs with the 10/90% wt PIM-1/6FDA-DAM blend containing (**a**) 1% wt, (**b**) 10% wt, and (**c**) 20% wt of ZIF-8; the MMMs with the 20/80% wt PIM-1/6FDA-DAM blend containing (**d**) 3% wt and (**e**) 10% wt of ZIF-8; and 90/10% wt PIM-1/6FD-DAM blend with (**f**) 10% wt of ZIF-8 and (**g**) its EDX mapping. (**h**) A scheme of the MMMs explaining the filler distribution is also included. (**i**) Gas separation performance of the PIM-1/6FDA-DAM blends for CO_2_/CH_4_ mixtures at 35 °C and 3 bar of feed pressure. Adapted from [86] Copyright © 2019, Elsevier.

With this mixture, they prepared MMMs by adding different amounts of ZIF-8, obtaining excellent results for the separation of CO_2_ over CH_4_ at 10% wt of the filler. In terms of overall performance, they reached P_CO2_ ~2900 and P_CH4_ ~108 Barrer with α_CO2/CH4_ ~26, which pushed their MMMs system far over the 2008 upper bound for CO_2_/CH_4_. The MMMs prepared with a higher ratio of PIM-1, apart from showing lower compatibility with 6FDA-DAM, showed an increase in the permeability (P_CO2_ ~10,000), but the loss in selectivity (α_CO2/CH4_ ~10) worsened the overall performance. Likely their most interesting conclusion, which is very important for this review, is that the notoriously low compatibility between ZIF-8 and PIM-1 [87,88] was improved by the addition of 6FDA-DAM, as assessed by SEM (Figure 6).

#### 2.2.2. Improvement in Permeability with Crystalline Fillers

**Figure 7:**
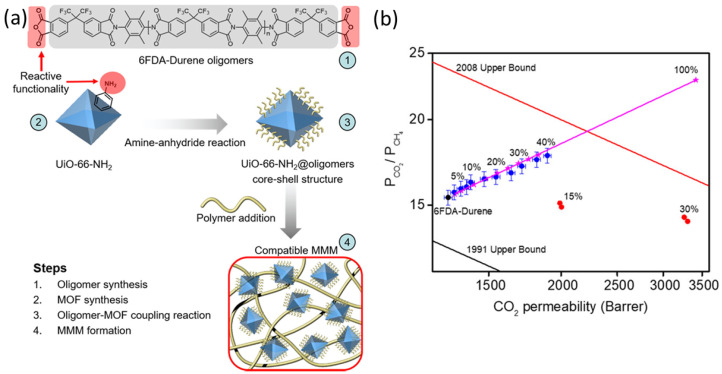
(**a**) Steps of Postsynthetic Modification of UiO-66-NH2 and Formation of MMMs. (**b**) Improvement of the permselective properties upon addition of post-synthetically modified UiO-66-NH_2_ into 6FDA–Durene polyimide (blue symbols) and prediction by the Maxwell model (pink line, Equation (2)) and comparison with the effect of the unmodified UiO-66-NH_2_ (red spheres). Adapted from [89] Copyright © 2020, American Chemical Society.

The improvements in selectivity just discussed in the previous section represent one way to increase the overall performance of MMMs for gas separation. As anticipated, for those applications that involve large gas volumes, it is important that in addition to high selectivity, the membranes also show high permeability, so the separation process becomes quicker and more commercially appealing. From this point of view, the best approach seems to prepare MMMs starting from already permeable matrices and combining them with appropriate fillers that would further increase the flux, while keeping similar selectivities. Because of their characteristically high porosity, inorganic fillers such as MOFs have been identified as ideal candidates also for this task. A good example is provided by the group led by Zachary Smith at MIT [89]. They used UiO-66-NH_2_ as a filler for MMM, exploiting the reactivity of the amino groups of this MOF that is known as having a remarkable affinity for CO_2_ [90,91]. To achieve enhanced interface compatibility between the MOF particles and the polymer matrix, they reacted the amino groups of the MOF with the anhydride ending groups of short oligomers of 6FDA–Durene polyimide, decorating the filler with a small part of the amorphous structure of the polyimide and generating a more homogeneous environment. In this way, a compatible CO_2_-selective MMM was obtained via post-synthetic modification of another CO_2_-selective one (Figure 7).

In this clever way, they produced defect-free MMMs with a maximum loading of 40% wt of the chosen filler, simultaneously addressing the typical compatibility problems between polymers and MOFs and improving the overall performance of the membrane. The initial performance improved from P_CO2_ ~1280, P_CH4_ 83 Barrer and α_CO2/CH4_ ~15.4 of the pure 6FDA-Durene polymer, to P_CO2_ ~1890, P_CH4_ 107 Barrer and α_CO2/CH4_ ~18 of their MMM system at 1 bar. The trend that brought their new material close to the 2008 upper bound could be very well described by the Maxwell model (Equation (2)). In addition, 50:50 CO_2_/CH_4_ mixed-gas permeation tests showed how upon increasing the total pressure from 2 to 30 bar, 6FDA-Durene film suffered from a significant reduction in selectivity, more than 50%, whereas the 40% MOF MMM retained its high selectivity. This filler increased MMM stability and enhanced the membrane plasticisation resistance [92], reducing the α_CO2/CH4_ by only 15% from 19.30 at 2 bar to 16.5 at 30 bar. The MMM also showed some resistance to ageing, with stabilisation performance up to 5 weeks after the initial measurement.

Often, compatibility problems can be solved with the addition of third components, such as ionic liquids (ILs) as described in a paper by Noble and co-workers [48]. In their work, it was shown that the addition of ILs to an MMM system, composed of cross-linked poly-ionic-liquid and SAPO-34 zeolite as an additive (up to 30% wt loading), not only improved the adhesion of the zeolite to the membrane but also enhanced the performance for the CO_2_/CH_4_ separation. The permeability increased from P_CO2_ ~9 to ~43 Barrer, with the best result at 20% wt loading, while the selectivity of CO_2_ over CH_4_ remained almost constant at ~30. 

**Figure 8:**
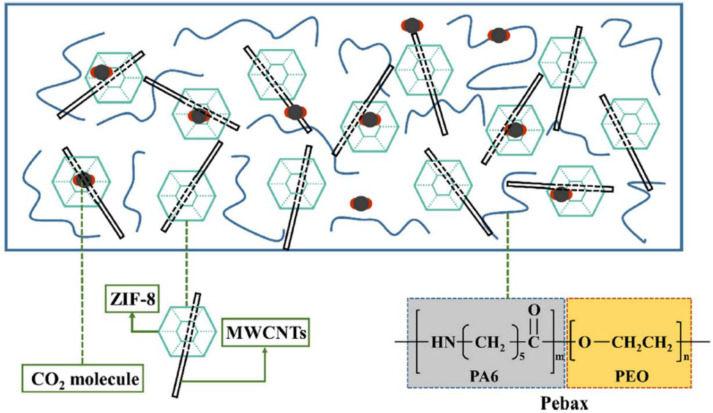
ZIF-8 and MWCNTs into Pebax^®^. Adapted from [93] Copyright © 2020, Elsevier.

Although it is known to suffer from compatibility issues with several soluble matrices, ZIF-8 is still one of the most used MOFs to prepare high-performing MMMs [41]. To overcome adhesion problems, Xue et al. inserted multiwalled carbon nanotubes (MWCNTs), as a third component in ZIF-8-based MMMs, with the aim of improving its adhesion to Pebax^®^1657 and, simultaneously, increasing its low permeability for CO_2_ [93]. Their MWCNTs@ZIF-8 particles were dispersed into a solution of Pebax^®^ (Figure 8) with loadings of up to 12% in weight. The obtained MMMs were characterised by SEM and TEM, which showed a homogeneous distribution of the filler that confirmed the desired increase in compatibility. The gas separation experiments resulted in an enhancement of the CO_2_ permeability, from the starting 144 to 186 Barrer, which was also coupled with an increase in the CO_2_/N_2_ selectivity from α_CO2/N2_ ~50 to ~61. The stronger affinity of the MOF for CO_2_ helped the MMM to surpass the 2008 upper bound and the authors explicitly attributed the improvement in performance to the better dispersion of the filler into the matrix, which prevented the typical aggregation of ZIF-8.

Aiming to improve the permeability of Matrimid^®^, Musselman and her group used MIL-53 as a filler for MMMs [94]. Moreover, this MOF is a well-studied framework, as most of its analogues are known to be able to change their morphology from an open-pore to a closed-pore framework in different conditions. This makes it a perfect example of what is now known as “breathing MOFs” [80,95], and for this reason, it seems an ideal filler to be used to prepare high-performing membranes. In their work, it was used in 33.3 and 37.5% wt loading to form MMMs with Matrimid^®^ via a sequence of sonication and priming, intended to prevent its typical agglomeration issues [96]. The permeability for CO_2_, N_2_ and H_2_ improved from P_CO2_ ~8.4, P_N2_ ~0.25 and P_H2_ ~26 to P_CO2_ ~40, P_N2_ ~0.42 and P_H2_ ~66, and these results were also coupled with an increase in selectivity α_CO2/N2_ that improved from ~34 to ~95 and α_H2/N2_ from ~103 to ~157. In an attempt to further increase the performance of PIM-1, Telfer et al. [57] employed their previously reported MUF-15, with a BET surface area of 1033 m^2^ g^−1^ [97], to prepare MMMs for CO_2_ separation. Interestingly, they succeeded in obtaining promising results with only 2–5% wt of the filler, which allowed them to minimise the typical compatibility problems at the interface with the matrix. Their system provided MMMs with P_CO2_ 36% higher than the original PIM-1 and with a very similar CO_2_/N_2_ selectivity (~19). This is a stronger increase than the Maxwell model predicts, suggesting other effects than simply adding a preferential path for gas transport. The good results obtained from pure gas measurements (only considering ideal separations) were confirmed with a 50/50% mixture of CO_2_/N_2_, which also showed slower ageing compared to neat PIM-1, although they tested it only 35 days after the first measurement. An excellent example that shows the potential issues caused by the lack of affinity between the crystalline filler and the amorphous matrix is highlighted by a paper from Deng et al. [98]. In this work, they added two-dimensional leaf ZIFs (ZIF-L-Zn and ZIF-L-Co) to a Tröger’s base (TB) polymer and used them to boost its hydrogen permeability. TB-PIMs are known for their good selectivity for H_2_, as Tröger’s base core is very rigid and enhances its separation from other gases by molecular sieving [99]. In this study, Deng and co-workers improved the permeability of the pristine polymer with filler loadings of up to 20% wt for both ZIF-L-Zn and ZIF-L-Co, improving from P_H2_ ~290 and P_CO2_ ~148 Barrer to P_H2_ ~1235 and P_CO2_ ~550 Barrer. However, compared to other entries in this review, they found a significant reduction in the selectivity over bulkier gases, such as N_2_ and CH_4_, as their permeability also increased after the addition of the filler. This unusual behaviour led to a reduction of selectivity α_H2/N2_ from 49 to 27 and α_H2/CH4_ from 50 to 25, compared to the neat membrane. Although the characterisation shows good compatibility between the fillers and matrix, the authors claim that interfacial voids between them cannot be completely avoided. If bulkier gases could pass through these microvoids, it is plausible to attribute to them the reduction in selectivity. Nevertheless, because of the good increase in permeability, the obtained data showed that these MMMs surpass the 2008 upper bounds. 

As a last example of this section, focused on the use of crystalline materials as filler for MMMs, in the attempt to improve both permeability and selectivity of PIM-1, Attfield et al. [100] prepared mixed matrix membranes with a dispersion of amine- and ethylene diamine-functionalized MIL-101 fillers. Comparison with MMMs made with the non-functionalised MIL-101 revealed changes in compatibility between the filler and the matrix. SEM images confirmed the lack of sedimentation of the functionalised filler into the membrane, which led to the production of homogeneously dispersed MMM. The enhanced compatibility allowed the membrane to accommodate the high loading of the different MOFs, which reached 28% wt. The permeability measurements showed a significant increase in the gas separation performance with the highest amount of the non-functionalised MIL-101, reaching P_CO2_ ~35,600 Barrer, which is in the range of the ultrapermeable PTMSP [101], also coupled with enhanced CO_2_/N_2_ selectivities (α_CO2/N2_ ~15) that pushed them far above the 2008 upper bound. Interestingly, there was no significant difference in performance with the two amino-functionalised MOFs, and they both produced lower permeabilities than the non-functionalised MIL-101, with P_CO2_ ~10,000 Barrer and α_CO2/N2_ ~20. These results showed that, despite the fact that the amino-functionalisation of the MOFs led to better filler-matrix compatibility, in this case, their reduced porosity (likely due to a pore-filling effect of the amino-groups) led to a reduction of the overall performance.

### 2.3. Advances in the Field of MMMs with Amorphous Fillers

As seen for the crystalline fillers, amorphous additives have also been extensively used to produce high-performing mixed matrix membranes to improve permeability, selectivity or both. Some of the most typical examples are graphene oxide [102], porous organic polymers (POPs) [103], porous aromatic frameworks (PAFs) [104] and polymers of intrinsic microporosity (PIMs) that, with the appropriate choice of the monomers and their functionalisation, often work as both the matrix and the filler [105,106]. One of the main advantages of having amorphous fillers is that compatibility with the equally amorphous matrix is normally improved.

#### 2.3.1. The Importance of Addressing the Potential Lack of Compatibility at the Interface between Filler and Matrix

Having exposed several potential (and often tangible) issues in terms of compatibility at the interface between crystalline fillers and amorphous matrices, and to avoid any associated drop in performance, researchers recently moved their attention to the utilisation of organic and amorphous additives to substitute the inorganic and crystalline ones. The main reason lies in the thought that the amorphous phase of the matrix would better blend with a similarly amorphous filler, in a sort of “like-dissolves-like” fashion [107]. As noted in the previous sections, in various works, researchers exposed poor compatibility between the matrix and filler that, often, resulted in sedimentation and aggregation of the additives [108], forming microvoids at the filler-matrix interface. This behaviour led to the worsening of the overall gas separation performances, often because of a drop in the selectivity as gases of all sizes could pass through these voids. 

**Figure 9:**
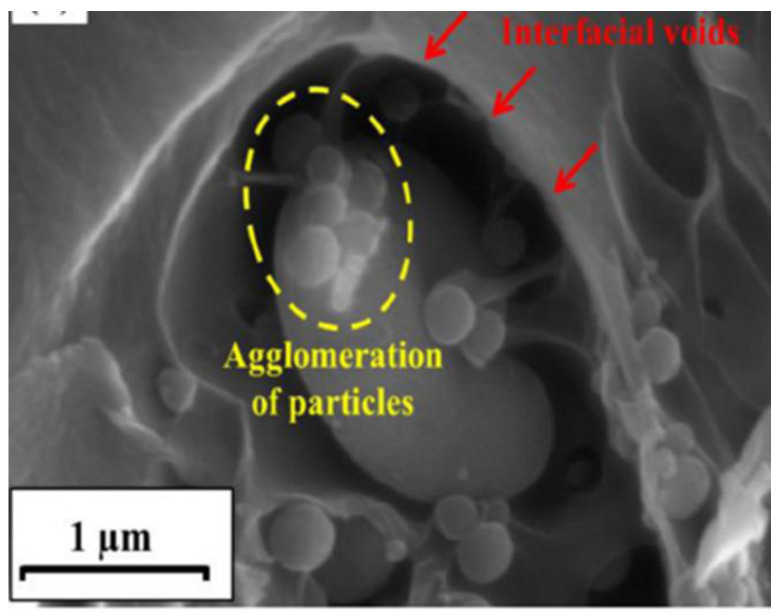
SEM cross-section images showing agglomeration of MCM-41 mesoporous silica dispersed in polyethersulfone. The poor compatibility between the polymer and the unmodified fillers causes the formation of microvoids and a dramatic change in performance. Adapted from [111] Copyright © 2016, Elsevier.

ZIF-8, for instance, is one of the most used MOFs for the preparation of high-performing MMMs [109], but in several cases, its compatibility with the soluble matrix was not adequate to achieve or maintain good separation performance. In many of the analysed articles, this problem was solved by functionalising it with extra organic moieties that afforded a better adhesion to the polymeric substrate. Steunou and co-workers [44] analysed in detail the affinity of ZIF-8 with two different polymers of intrinsic microporosity, PIM-1 and PIM-EA-TB [99], testing it via both molecular simulations and experimental studies [110]. Firstly, they synthesised and characterised ZIF-8 following known procedures to be sure they obtained results consistent with the literature. Then, to measure the potential compatibility at the interface, they analysed the interactions between the MOF and the surface of the two polymers by Radial distribution functions (RDFs), highlighting regions where they identified potential “*interfacial microvoids*”, in both PIM-EA-TB and PIM-1 based MMMs. The experimental results confirmed this hypothesis with the preparation of a series of MMMs with the two PIMs and ZIF-8 (at loading between 10 and 50% wt), showing that sedimentation and aggregation issues do exist, as proven via a combination of different TEM techniques. Another example that aimed to address this potential problem comes from a paper published by Sadeghi and co-workers [111], who analysed in detail the compatibility at the interface between polyethersulfone and dispersed MCM-41 mesoporous silica. SEM observations showed that, despite producing seemingly homogenous membranes, the dispersion of the inorganic filler increased the roughness of the final material, creating microvoids and drastically changing the performance with loading of up to 20% wt (Figure 9). To prevent the formation of these defects, they modified the surface of the silica anchoring aminopropyl groups. The organic moieties enhanced the adhesion and prevented loss of performance and robustness. Similar work was recently published by Barooah et al. [112], who used the same strategy to improve the CO_2_ separation performance of a PVA/PEG/PEI/TETA blend membrane by incorporating amino-functionalised silica nanoparticles (SNPs) as a filler. The inclusion of the modified SNP not only improved the permselectivity but also the overall robustness of the matrix membrane.

An accurate analysis of previous works [44,58,106,107] suggests that using purely organic and amorphous fillers would, indeed, lead to significant improvements in terms of compatibility between soluble and insoluble components of the MMMs. For this reason, the next section of this review aims to discuss different examples where authors used completely amorphous and organic fillers, preferring them over their seemingly high-performing crystalline counterparts. As performed for the crystalline fillers, in the next parts, the improvements in selectivity and permeability for these MMMs will also be discussed separately.

#### 2.3.2. Improvement in Selectivity with Amorphous Fillers

**Figure 10:**
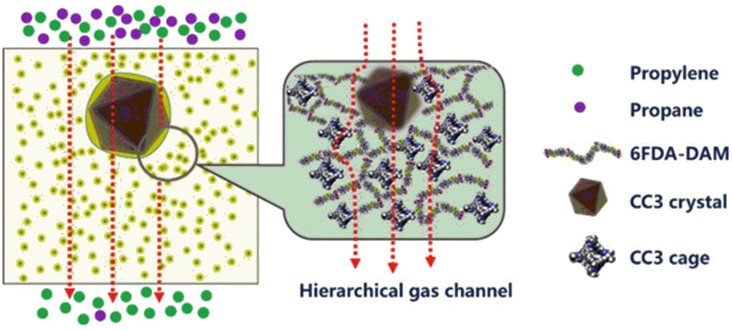
Gas channels in MMMs of 6FDA-DAM with recrystallized CC3. Adapted from [66] Copyright © 2020, Elsevier.

In a recent paper, the group of Wanqin Jin [66] described the use of CC3 molecular cages [113] as fillers for MMMs, in combination with 6FDA-DAM as the soluble matrix. These cages are normally crystalline [65], but the framework is entirely organic, so they actually represent an ideal transition in the analysis of these two morphologies. The obtained MMMs were used for the separation of propylene (C_3_H_6_) from propane (C_3_H_8_). The Jin group discovered that the use of these organic fillers created hierarchical channels in the membranes that favour the separation of the two gases and, most importantly, the morphology analysis of the MMMs confirmed the production of defect-free membranes, showing a highly homogeneous dispersion of the filler into the matrix of up to 30% wt (Figure 10). 

The inclusion of the CC3 molecular cage achieved the best results at 20% wt loading as further amounts, despite being well-anchored in the system, produced a sharp decrease in selectivity. The latter, as seen in other examples, may hint at the formation of microvoids that may also allow larger gases to diffuse in the membrane. The best permeability of the set reached P_C3H6_ ~480 Barrer, with a selectivity over C_3_H_8_ that was improved by up to α ~15.4 from the original ~6.4.

**Figure 11:**
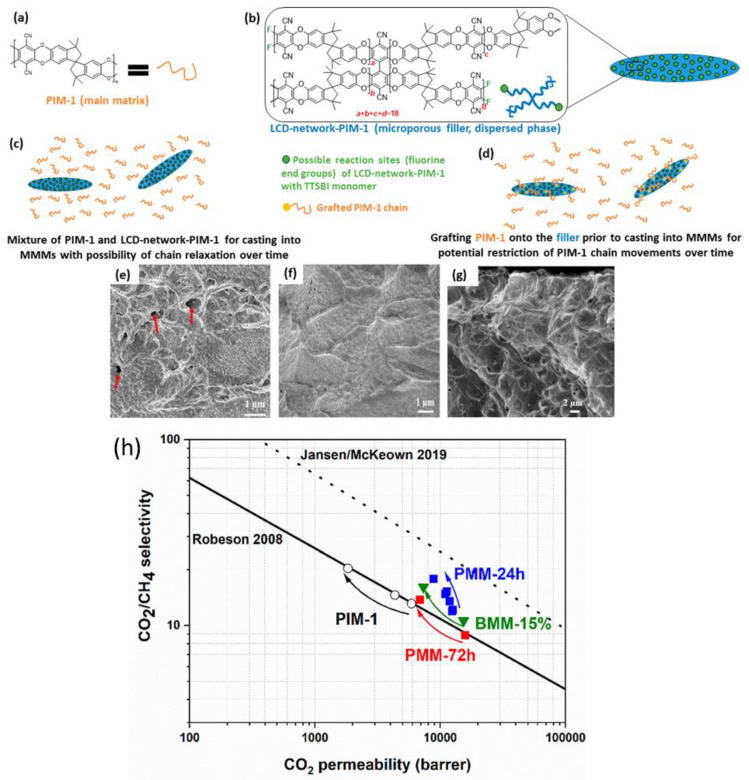
(**a**) Chemical structure of PIM-1, (**b**) chemical structure of low cross-link density (LCD) network-PIM-1, (**c**) representation of blend mixture of PIM-1 and LCD-network-PIM-1, (**d**) representation of PIM-1-grafted onto LCD-network-PIM-1. SEM cross-section images of (**e**) a MMM of PIM-1 with the 15% wt of LCD-network-PIM-1 (BMM-15%), with red arrows showing possible defects formed between the two phases, and (**f**,**g**) MMMs prepared via polymerization of PIM-1 in the presence of LCD-network-PIM-1. (**h**) Robeson plot of MMMs containing the ageing data of 15% wt LCD-network-PIM-1 (PMM-24 h, PMM-72 h and BMM-15%) and PIM-1 membranes for the separation of CO_2_/CH_4_. Adapted from [105] Copyright © 2020, American Chemical Society.

Another amorphous filler that is often functionalised with organic groups, with the aim of improving its performance, is graphene oxide (GO). In recent work, Gorgojo et al. [114] reported the preparation of MMMs with both non-functionalised GO and (3-aminopropyl) triethoxysilane-GO (APTS-GO). They were embedded into PIM-1 to form ageing-resistant MMMs. In this work, the authors used a different approach to the typical MMM formation, synthesising PIM-1 onto the surface of the graphene derivatives to then disperse these fillers into a more concentrated solution of PIM-1 to form the final MMMs. A comprehensive membrane characterisation by XPS, SEM and TEM analysis showed a very homogeneous distribution of the filler with no observable pinholes and no agglomeration, which demonstrated the high compatibility between the two compounds. The permeability measurements recorded a reduction of P_CO2_ compared to neat PIM-1, but this was counterbalanced with a commensurate increase in selectivity towards CO_2_/N_2_ and CO_2_/CH_4_, which led to an improvement in the overall performance. Most importantly, the combination of these two amorphous components seemed to promote anti-ageing behaviour, with a drop in CO_2_ permeability of only 10% over 150 days, with the best performance reached with 10% wt of APTS-GO. In another effort aimed to improve the general performance of PIM-1 and to stabilise it against ageing, the group of Peter Budd from the University of Manchester reported a filler derived from the main monomer used to synthesise neat PIM-1 (the spiro-biscatechol) [83], combining it with another poly-fluorinated monomer that formed an insoluble networked polymer [106]. This new amorphous filler produced nanosheet-like structures, with high BET surface area (940 m^2^ g^−1^, compared to 780 m^2^ g^−1^ of the original PIM-1 [83]). The loading of the new additive into a solution of PIM-1 led to the formation of MMMs in a wide range filler concentration between 0.5 and 10% wt and, surprisingly, they obtained the best performance with only 0.5% wt of the filler in terms of permeability, but with 10% loading in terms of selectivity (+46%). With this amount, they reported an increase in CO_2_ permeability (P_CO2_ ~9800, compared to ~5900 Barrer of the original PIM-1) and, most importantly, with selectivities over N_2_ and CH_4_ of α_CO2/N2_ ~15.9 and α_CO2/CH4_ ~14.4 that pushed them over the respective 2008 upper bounds. The new MMMs also showed good resistance to ageing, with only a small change in permeability and selectivity in 7 months (~35%). In a follow-up paper, the same group published a similar networked filler, but it was grown as an integral part of the polymer producing a sort of “in situ” mixed matrix membrane [105]. They obtained it by combining in the same reaction flask the monomers that form the insoluble filler (up to 15% wt) and the two monomers that produce PIM-1. By playing with the stoichiometry of the components and the calculated amount of the ending groups of the filler, it allowed a certain degree of cross-linking that further rigidified the polymeric chains but, at the same time, kept it soluble enough to cast a low cross-link density (LCD) membrane (Figure 11). In this clever way, compared to the previous work where they just blended the two components in the typical mixed matrix membrane fashion (filler dispersion in a solution of the matrix), they improved the overall performance of CO_2_/N_2_ and CO_2_/CH_4_ separations, approaching the CO_2_/CH_4_ 2019 upper bound [17] (P_CO2_ ~12,500-Barrer α_CO2/CH4_ ~11.9). The further increase in the compatibility between the filler and the matrix, which afforded virtually defect-free MMMs, also translated into further improved ageing resistance. The results showed only a 29% loss of the original CO_2_ permeability after >100 days, proving that the enhanced affinity between the filler and the matrix also helped to stabilise the performance. 

**Figure 12:**
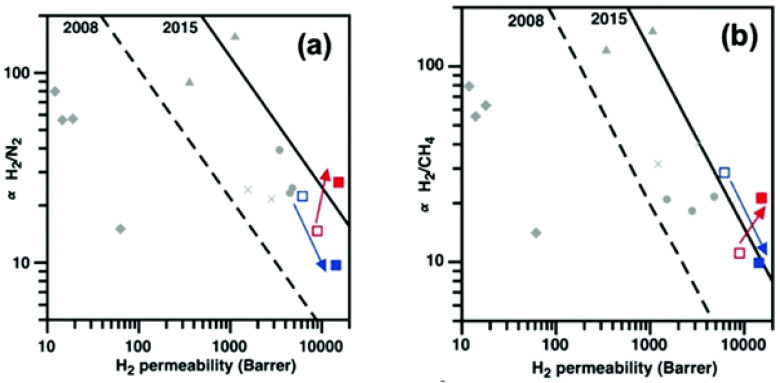
Robeson plots for (**a**) H_2_/N_2_ and (**b**) H_2_/CH_4_ for PIM-EA(Me_2_)-TB (□), PIM-EA(Me_2_)-TB/PAF-1 (▪), PIM-EA(H_2_)-TB (□), and PIM-EA(H_2_)-TB/PAF-1 (▪) PAF-1 into PIM-EA-TB. Adapted from [116]. Copyright © 2020, RSC.

Compared to amorphous polymers, crystalline MOFs often show much higher BET surface areas, so they facilitate more efficient gas diffusion and transport. The group led by Matthew Hill at CSIRO in Melbourne found a brilliant alternative with the use of a porous aromatic framework (PAF-1) as a filler. This is an amorphous polymer published several years before that holds an astonishing Langmuir surface area of ~7000 m^2^ g^−1^ [115]. In one of their several works with this highly porous polymer, they mixed it with the ultrapermeable PTMSP [101] increasing the CO_2_ permeability up to 25% more than the original polymer, and then with PIM-1 [83], reporting a massive 320% permeability increase in the same gas. To better understand how the filler influences the performance, they studied the compatibility between PAF-1 and PIM-1 by SAXS/WAXS and solid-state ^13^C NMR, realising that the pendant methyl groups on the PIM-1 backbone have a crucial effect on both enhancing performances of the MMM and stabilising it towards physical ageing (up to 240 days). Their best results afforded defect-free MMMs with up to 10% wt loading of PAF-1. Interestingly, they noticed that the permeability of larger gases (N_2_ and CH_4_) decreased with time, while the permeability of CO_2_ remained almost constant (less than 7% loss in 240 days). This, of course, translated into a large increase in CO_2_/N_2_ selectivity upon ageing. Using PAF-1 with another highly permeable PIM, this time the PIM-EA-TB [99], Lau et al. applied the same concept and confirmed that the presence of methyl groups on the soluble polymeric backbone translated into an enhanced affinity for PAF-1 [116]. To obtain a final confirmation, they prepared MMMs with PAF-1 and both a methyl containing PIM-EA-TB [99] and completely analogous (PIM-EA(H_2_)-TB, which does not have pendant methyl groups in the backbone [117]. The unique behaviour of PAF-1 in combination with anchoring methyl groups showed that the MMM formed with PIM-EA(H_2_)-TB (again with loading of up to 10% wt), aged in the same way as the original polymer, with a sharp decrease in permeability over time coupled with a commensurate increase in selectivity that keeps it in the same region of the H_2_/N_2_ and H_2_/CH_4_ upper bounds (Figure 12). Conversely, the one made with methyl-containing TB-PIM aged more gracefully. In the overall performance assessment, they observed a ~70% increase in H_2_ permeability for both methyl and methyl-less MMMs, and a decrease in N_2_ and CH_4_ permeability over time of ~5 and ~11%, respectively, but the methyl-less TB-PIM held the performance for much longer. As for the example with PIM-1, this led to an increase in the selectivities that pushed the overall performance well over the 2015 upper bounds for H_2_/N_2_ and H_2_/CH_4_ [16]. Another interesting feature that they connected with the use of PAF-1 is that, along with the same anti-ageing behaviour found for PIM-1, the TB-PIM-based MMMs also showed better plasticization resistance, confirming the great affinity between the matrix and this amorphous filler.

As anticipated, a clear advantage in favour of the amorphous fillers lies in the versatility of their synthesis, which permits post-polymerisation modifications of the structures more easily than for crystalline materials. This feature gives the option to start from non-functionalised backbones and to tune the selectivity towards important gas pairs with the introduction of extra organic moieties. This pathway is far more complicated with MOFs, where the functional groups must be necessarily introduced to the monomers before the formation of the framework. A good example is provided by the functionalisation of PAFs by Hill et al. [104], who prepared MMMs with aminated and sulfonated versions of PAF-1, starting from the correspondent hydrocarbon polymer [118,119]. Compared to the original PAF-1, with the addition of the functional groups, the porosity of the new PAFs decreased, most likely because of their pore-filling behaviour and higher cohesive forces between the polar functional groups. In fact, the BET surface areas decreased from ~7000 m^2^ g^−1^ of the original PAF-1 to 900 m^2^ g^−1^ for aminated PAF and ~830 m^2^ g^−1^ for the sulfonated versions. Nevertheless, the new organic fillers with loadings of up to 10% wt were added to a solution of PTMSP to obtain high-performing MMMs. Despite the sharp loss of porosity, which was expected to slow down the gas diffusion and transport, the enhanced affinity of the functional groups for CO_2_ led to an increase in their solubility coefficients, which resulted in an overall improvement of the permeability (85% more than the original PTMSP). Furthermore, in this case, along with the enhanced compatibility with the soluble matrix, the functionalised organic fillers provided an improvement in the ageing resistance. The collaboration between the CSIRO group and Ingo Pinnau at KAUST produced a mixed matrix membrane with a combination of PAF-1 (5–10% wt) [120] and TPIM-2 [121]. Despite the great affinity between the matrix and the filler, as evaluated by SEM, they did not report the same entanglement between the two components that they noticed for other PIMs/PAF-1-based MMMs [104,116]. Even so, permeability measurements showed a 196% increase in P_H2_, compared to neat TPIM-2, which reached up to P_H2_ ~5000 Barrer. This was coupled with an enhancement of the H_2_/N_2_ selectivity, which improved from α_H2/N2_ ~19 to ~27. 

**Figure 13:**
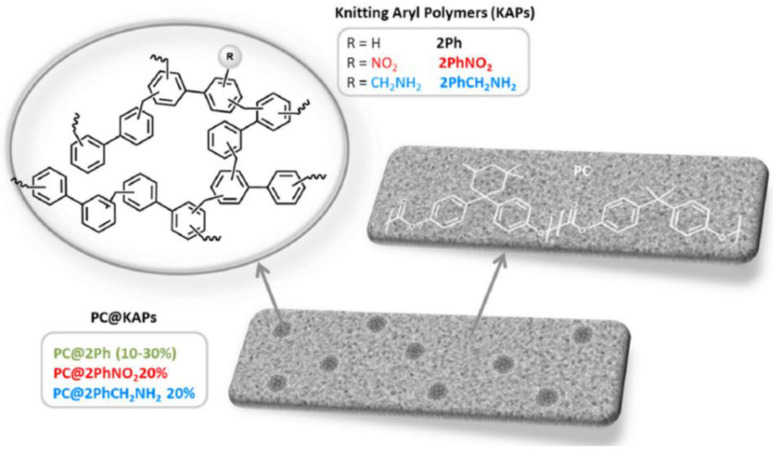
Schematic representation of porous organic polymers as fillers into polycarbonate polymers. Adapted from [122] Copyright © 2020, Elsevier.

As seen for the crystalline fillers, the effect of the addition of porous polymers is particularly important when used to further improve the gas selectivity of low-permeability membranes. In very recent work, Maya et al. [122] reported the inclusion of porous organic polymers as fillers into polycarbonate polymers. The latter are interesting materials as they are cheap and readily available, but the main drawback is their low permeability (P_CO2_ ~17.6, P_H2_ ~21.4, P_CH4_ ~0.9), which is not counterbalanced by a high enough increase in selectivity. To improve these performances, the authors prepared fillers consisting of different hydrocarbon-based polymers (Figure 13), that were functionalised with nitro and amino groups. To afford a better dispersion, the authors observed that long sonication times were required. This is an important suggestion, which is often reported in other papers and improves the dispersions of both crystalline and amorphous fillers in MMMs [123,124,125].

The longer sonication times afforded a more homogeneous dispersion of the filler, which translated into a better interaction with the matrix that produced an apparent entanglement of polycarbonate chains into the pores of the fillers. This combination contributed to the stabilisation of the mixed matrix membrane and, despite the fact that the pore-filling effect of the polymer chains can reduce the gas transport (as seen for other works [46]), the permeability of the polycarbonate layer still benefited from the inclusion of the amorphous porous materials. Indeed, with loadings between 10 and 30% wt, the permeabilities reached P_CO2_ ~52, P_H2_ ~62 and P_CH4_ ~2.7, without a significant change in selectivity. The addition of the functionalised fillers (-NO_2_ and -CH_2_NH_2_), in contrast, did not produce a substantial increase in the permeability but improved the selectivity instead, improving it from the initial α_CO2/CH4_ 19.6 and α_H2/CH4_ 23.8 to 24 and 34, respectively.

#### 2.3.3. Improvement in Permeability with Amorphous Fillers

As considered for MOFs and other crystalline filler-based MMMs, the use of appropriate additives frequently helps to increase the permeation rate and separation of commercially important gas pairs, without losing too much in selectivity. This is crucially important, as often pointed out, because more permeable materials increase the flux and allow the treatment of larger gas volumes, reducing the contact time with the membrane. For the same reasons discussed in the crystalline materials section, the most evident improvement in permeability due to the insertion of appropriate fillers is often obtained by combining them with polymeric membranes that start from a low flux. In this way, researchers aim to accelerate gas permeation while maintaining good selectivity that affords the necessary gas purity. A good example is given by the group of Cristina Alvarez [126], who prepared a triptycene-isatin porous material and used it as a filler to create new MMMs with, respectively, the low-permeability and commercial Matrimid^®^ and two other (novel from their work) linear polyimides, namely 6FDA-6FpDA and 6FDA-TMPD. The porous network blended very well with all the matrices with a filler loading of up to 30% wt, providing transparent MMMs with no evident signs of agglomeration of the filler as proven by WAXS and SEM. As seen in other work, the study of the sonication time, necessary to ensure better dispersion of the filler into the matrix solution, was revealed to be crucial to obtain the best blending results. In terms of performance, they found that high amounts of filler produced a corresponding increase in permeability, improving the CO_2_ permeability between 2.5- and 7-fold compared to neat membranes, and approaching the CO_2_/N_2_ 2008 upper bound as they retained most of the selectivity over N_2_. 

**Figure 14:**
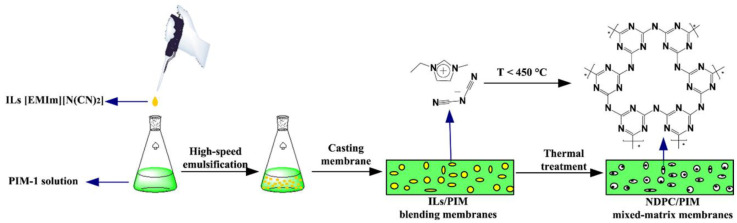
PIM-1-based mixed matrix membranes with methylimidazolium as ionic liquid (IL) as a filler. Adapted from [127] Copyright © 2018, Elsevier.

In recent work, Gascon et al. [55] synthesised two different benzimidazole-linked polymers (BILPs) that were used to enhance the performance of Matrimid^®^-based MMMs. The addition of the porous filler produced very good separation performance with loading between 8 and 24% wt, and the compatibility between the two amorphous components proved excellent, which is almost a constant for these amorphous filler-based MMMs. The porosity of the benzimidazole fillers gave a boost to the permeability results at the highest loading (24% wt), with a ~3-fold improvement in P_CO2_ without any loss of selectivity over N_2_. The importance of these results is clear from the excellent separation performance of a 15:85 CO_2_/N_2_ gas mixture at 308 K, which represents almost ideal post-combustion CO_2_ capture conditions. Ding and co-workers showed the use of triazine-based porous carbons as fillers for PIM-1-based mixed matrix membranes [127]. To enhance the performance and the compatibility between filler and matrix, they cast a film of PIM-1 in the presence of 2–10% wt of 1-ethyl-3-methylimidazolium as an ionic liquid (IL) and then by thermal treatment of the resulting MMM, they forced thermal cross-linking of the IL and produced in situ 3D nitrogen-doped porous carbons (NDPC, Figure 14). In addition to being used as the main component, ionic liquids are also interesting additives for mixed matrix membranes [128]. They were already discussed in this review in the crystalline MMMs part, as they were successfully used to improve the compatibility of MMMs with zeolites as reported by Noble [48]. The result that the Ding group obtained for their MMMs at 2–5% wt loading demonstrated an impressive improvement in performance, showing an exceptional increase in CO_2_ permeability with P_CO2_ ranging from 32,000–40,500 Barrer (from the initial 4000 of the neat membrane), at the expense of a relatively low loss of selectivity (α_CO2/N2_ decreased from 16 to 12). This enhancement allowed their MMMs to surpass the 2008 upper bound. A very recent paper reported a special case, where ionic liquid was the major component in the MMM, and where a POP was dispersed in a so-called ion-gel mixed matrix membrane, based on a poly (ethylene glycol) diacrylate (PEGDA) network with up to 80% wt of ionic liquid [129]. They are not as permeable as PIM-based MMMs but can reach remarkably high selectivities, with PCO_2_ up to 91 Barrer (from the starting 62 of the neat membrane) and α_i_ (CO_2_/N_2_) = 53 from the starting 17).

The triazine scaffold also proved very useful for producing high-performing MMMs for Janiak’s group. In very recent work, they prepared a triazine-fluorene network polymer that they used as a filler in combination with Matrimid^®^ and polysulfone, increasing their low permeability while maintaining good selectivity [130]. The high surface area of the filler (~760 m^2^ g^−1^) helped in boosting the overall permeability by increasing the diffusivity of small or more condensable gases. This combination allowed them to obtain remarkable results for the CO_2_/CH_4_ separation. The addition of amorphous fillers showed enhanced compatibility at the interface between the matrix and the filler, as the Janiak group did not find any defects in their structures by SEM analysis, as only above 24% wt loading did they start to see significant signs of agglomeration. In terms of separation performance, P_CO2_ increased from 5.4 and 6.8 Barrer for the neat polysulfone and Matrimid^®^ membranes, respectively, to 12.8 and 17.8 Barrer for the corresponding MMMs (best results at 24% wt loading), whereas the selectivity over CH_4_ remained constant at ~30 and ~44, respectively. 

**Figure 15:**
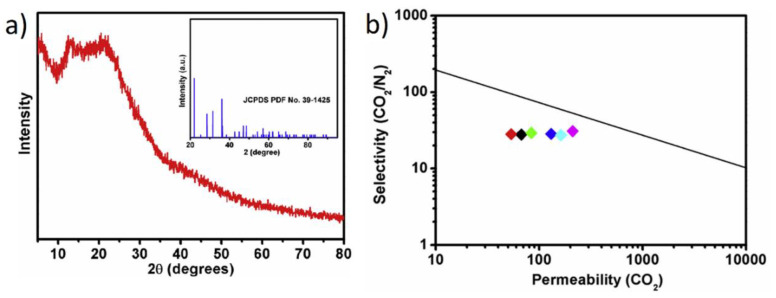
(**a**) XRD patterns of the ODPA-TFMB powders, with modified silica nanoparticles (insert). (**b**) Robeson plot of CO_2_/N_2_ separation for the MMMs with different loadings of filler: (◆ pure membrane, and MMMs with 0.3% (◆), 0.6% (◆), 0.9% (◆),12% (◆) and 20% (◆) of filler). Adapted from [131] Copyright © 2019, Elsevier.

Hu et al. [131] modified silica nanoparticles with amino groups and then tested them as fillers in combination with a benzidine-based polyimide (ODPA-TFMB). This example is particularly relevant for this review, as they turned a crystalline material into a completely amorphous one (Figure 15a). They found that this transformation not only improved the blending of the filler into the membrane but it also increased the diffusion and the gas transport for specific gas molecules. The MMMs were prepared at loadings between 3 and 50% wt, and the amino groups showed the expected affinity between the matrix and the filler, with no obvious defects. The MMMs showed a 3-fold increase in the CO_2_ permeability, from P_CO2_ ~67 of the neat membrane to 210 Barrer at 20% wt of the filler, with a constant CO_2_/N_2_ ideal selectivity of ~30, close to the 2008 upper bound (Figure 15b). Interestingly, the results further improved with a 15/85 CO_2_/N_2_ gas mixture close to post-combustion CO_2_ capture conditions [55]. Zhang et al. prepared a series of mixed matrix membranes with aminosilane-functionalized graphene oxide as fillers, aiming to address the potentially poor compatibility at the interface between the matrix and filler in MMMs [132]. They decided to use Pebax^®^1657 as the matrix and, surprisingly, the best results came with only 0.7% wt of the filler, with an increase in P_CO2_ from ~96 to 150 Barrer coupled with CO_2_/N_2_ selectivity of ~45 (their values of neat Pebax^®^1657 showed P_CO2_ ~96.2 and α_CO2/N2_ ~39.1). As seen for other amorphous filler/amorphous matrix combinations, the addition of the filler also improved its mechanical properties. Interestingly, they tested the performance of their MMMs using a humid 20/80 CO_2_/N_2_ mixture, finding that the performance for this separation further increased in these conditions. In fact, with only 0.9% wt loading, they reached a remarkable P_CO2_ ~934 Barrer. The extra boost given by these fillers is most likely due to the alkalinity of the amino groups, which allow more efficient binding of CO_2_ in the presence of water, because of the formation of carbonic acid.

**Figure 16:**
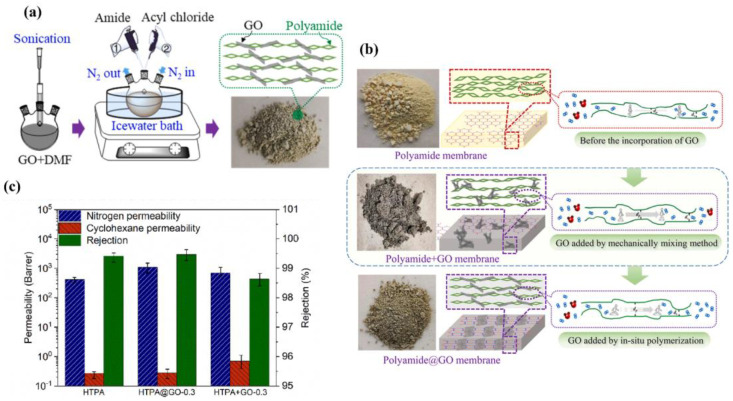
(**a**) Synthesis procedure of polyamide@GO starting from 2,6,14-triamonotriptycene (Trip) -based polymers and graphene oxide (GO). (**b**) Schematic transport channels for nitrogen permeation through the membranes. (**c**) Comparison of separation performance for pure polymer and polyamide@GO and polyamide + GO membranes. Adapted from [102].

In another effort aimed to improve permeability and the compatibility between the matrix and the fillers, Mao et al. prepared a range of polyamide graphene oxide (PA-GO) based MMMs and evaluated their performance for the separation of cyclohexane/nitrogen mixtures [102]. In this work, an in situ polymerization between 2,6,14-triamonotriptycene (Trip), octanedioyl chloride and graphene oxide (GO) was successfully performed for the preparation of polyamide@GO mixed matrix polymers with different GO contents (from 0.15 to 0.6% wt) (Figure 16a). 

A pure polyamide membrane [133] and the mechanically mixed polyamide + GO membranes were also investigated for comparison. The incorporation of GO sheets was suggested to disrupt the efficient stacking of polyamide chains, increasing the amorphous domain and the average interlayer spacing, reflected in a higher BET surface area, and allowing for more transport channels for nitrogen permeation through the membranes (Figure 16b). The best performance was obtained using polyamide@GO 0.3% wt, where the permeability increased from 427 to 1098 Barrer with a rejection of 99.4% (Figure 16c). The lowest rejection for the mechanically mixed polyamide + GO membrane was explained by the aggregation of GO sheets, resulting in the formation of nonselective voids.

### 2.4. Practical Notes

For the final application of a membrane system, the priority is high permeability if large gas volumes must be separated, or high selectivity if a high purity of the final product is required. In this light, the data discussed in the previous sections are summarized in Table 1, highlighting the main effect of the filler on either the permeability or the selectivity.

## 3. Conclusions and Outlook

New mixed matrix membrane (MMMs) systems are employed by an increasing number of research groups to improve the separation and purification of commercially important gases from mixtures. Along with the improvement in the overall performance of the neat polymeric membranes, the inclusion of appropriate fillers also aims to generate anti-ageing and anti-plasticisation resistance. In this review, we analysed and discussed the difference in the performance of several of these composite systems, especially focusing on the nature and morphology of the fillers. Particular attention was paid to addressing the main advantages and disadvantages when employing either crystalline additives (primarily MOFs as they are the most common) or their amorphous counterparts. In general, for both classes of fillers, it was observed that high porosity typically helps to increase the flux of initially low-permeability membranes, and this is typically due to the boost in the diffusion of small gases (i.e., H_2_) or the increase in the solubility coefficients for more condensable ones (i.e., CO_2_). When the fillers were adorned with organic functional groups, their addition often resulted in enhancing the selectivities for different gas pairs. The most important trend found in this review is that, despite both crystalline and amorphous additives successfully being used to boost permeability and selectivity for different gases, the amorphous fillers demonstrated enhanced compatibility at the interface between the matrix and the filler, which gives them a slightly better outlook when compared to the crystalline counterparts. According to several of the reported examples, this seems primarily due to the stark contrast in morphology between the rigid and inorganic surface of the crystalline fillers and the organic structure of the amorphous matrix. This problem leads to the creation of defects and microvoids that end up worsening performances, especially at a high loading of the fillers, but in addition, it seems to compromise the mechanical strength and robustness of the MMMs. The latter factor is especially considered crucially important for the stability of these hybrid systems, as the introduction of additives is known to disrupt the packing of the polymeric chains of the matrix and if the blend is not homogeneous, it translates into a weaker interaction at the interface, which makes the membranes more fragile. Amorphous fillers, instead, demonstrated a great affinity for the organic matrix that, in the end, constitutes a major part of the MMM (typically between 10 and 30% wt, with a few exceptions). If the two phases blend homogeneously, the result is a better adhesion of the filler to the matrix, which leads to the formation of virtually defect-free membranes. As often reported, this combination also imparts to these composite systems anti-ageing and anti-plasticisation properties. Molecular simulations and effective medium approaches or resistance models provide a fundamental understanding of the transport phenomena in relation to the MMM characteristics and are essential tools for the successful development of novel MMMs.

From this analysis, it could be projected that the use of amorphous fillers may have a bright future in the preparation of new MMMs. Nevertheless, the constant advancement in research on MOFs and other crystalline porous materials guarantees that they will always play an important role in the development of membranes for gas separation, especially with the preparation and tuning of new frameworks that contain more organic functional groups.

## Figures and Tables

**Table 1:** Improvement in selectivity and permeability with crystalline and amorphous fillers.

**Mixed Matrix Membranes with Crystalline Fillers**					
**Matrix**	**Filler ^a^**	**Type**	**Selectivity Improvement ^b^**	**Main Gas Pairs ^c^**	**Ref.**
Matrimid^®^ 5218	MIL-101 (Cr) (10%)	MOF	+62%	CO_2_/CH_4_ and CO_2_/N_2_	[75]
Matrimid^®^ 5218	Al-fum (20%)	Mesoporous	+63%	CO_2_/CH_4_ and CO_2_/N_2_	[77]
Matrimid^®^ 5218	NH_2_-MIL-53(Al) (40%)	MOF	+100%	CO_2_/CH_4_	[80]
PIM-1	UiO-66-NH_2_ (10%)	MOF	+35%	CO_2_/N_2_	[81]
Durene COOH-PI	UiO-66(Zr)-NH_2_ (10%)	MOF	+59%	CO_2_/CH_4_	[85]
PIM-1 + 6FDA-DAM	ZIF-8 (10%)	MOF	+30%	CO_2_/CH_4_	[86]
**Matrix**	**Filler ^a^**	**Type**	**Permeability Improvement ^b^**	**Main Gases ^c^**	
6FDA–Durene	UiO-66-NH_2_ (40%)	MOF	+60%	CO_2_, CH_4_ and N_2_	[89]
Pebax^®^-MH-1657	MWCNTs@ZIF-8 (12%)	Nanotubes	+30%	CO_2_, CH_4_ and N_2_	[93]
Matrimid^®^ 5218	MIL-53	MOF	+102%	CO_2_, CH_4_ and N_2_	[94]
PIM-1	MUF-15 (2–5%)	MOF	+36%	CO_2_ and N_2_	[57]
TB-PIM	ZIF-L-Zn (20%)	MOF	+271%	CO_2_ and H_2_	[98]
PIM-1	MIL-101 (47%)	MOF	+178%	CO_2_, CH_4_ and N_2_	[100]
**Mixed Matrix Membranes with Amorphous fillers**					
**Matrix**	**Filler ^a^**	**Type**	**Selectivity Improvement ^b^**	**Main Gas Pairs ^c^**	**Ref.**
6FDA-DAM	CC3 (20%)	Organic cage	+140%	C_3_H_6_/C_3_H_8_	[66]
PIM-1	APTS-GO (10%)	Graphene oxide	+27%	CO_2_/CH_4_	[114]
PIM-1	Networked PIM-1 (0.5–10%)	Network polym.	+46%	CO_2_/CH_4_	[106]
PIM-EA-TB	PAF-1 (10%)	Porous framew.	+80%	H_2_/N_2_ and H_2_/CH_4_	[116]
TPIM-2	PAF-1 (5%)	Porous framew.	+42%	H_2_/N_2_ and H_2_/CH_4_	[120]
PC	PhCH_2_NH_2_ (20%)	Porous framew.	+43%	H_2_/CH_4_ and CO_2_/CH_4_	[122]
**Matrix**	**Filler ^a^**	**Type**	**Permeability Improvement ^b^**	**Main Gases ^c^**	
6FDA-6FpDA	Triptycene-isatin (30%)	Porous framew.	+170%	CO_2_ and N_2_	[126]
Matrimid^®^ 5218	BILPs (24%)	Porous framew.	+181%	CO_2_ and N_2_	[55]
PIM-1	NDPC (5%)	Porous framew.	+650%	CO_2_ and N_2_	[127]
PEGDA	POP (0.5%)	Network polym.	+47%	CO_2_, CH_4_ and N_2_	[129]
Matrimid^®^	Triazine-fluorene (24%)	Porous framew.	+161%	CO_2_, CH_4_ and N_2_	[130]
ODPA-TFMB	Silica nanoparticles (20%)	Mesoporous filler	+213%	CO_2_ and N_2_	[131]

^a^ The percentage in brackets refers to the loading of the filler that produces the most improved performance after its addition. ^b^ Refers to the improvement of the performance compared to the pristine matrix polymer. ^c^ Gas pairs that produced the most improved performance after the addition of the filler.

## Data Availability

Not applicable.
